# Low‐κ Extension Doping for High‐Performance Carbon Nanotube Transistors: Toward High‐Speed, Energy‐Efficient Electronics

**DOI:** 10.1002/advs.202505543

**Published:** 2025-06-05

**Authors:** Hsin‐Yuan Chiu, Chen‐Han Chou, Guan‐Zhen Wu, Han‐Yi Huang, Bo‐Heng Liu, Chi‐Chung Kei, Chao‐Hsin Chien

**Affiliations:** ^1^ Institute of Electronics National Yang Ming Chiao Tung University Hsinchu 30010 Taiwan; ^2^ Taiwan Instrument Research Institute National Applied Research Laboratories Hsinchu 30076 Taiwan

**Keywords:** carbon nanotubes, low‐dimensional materials, low‐κ extension doping, metal–oxide–semiconductor field‐effect transistors

## Abstract

Carbon nanotubes (CNTs) present considerable potential as next‐generation logic switches owing to their ultra‐thin structure and exceptional electrical properties. Although recent advancements have achieved impressive direct current (DC) performance in individual devices, research on the architectural design of CNT‐based transistors remains limited. This gap is critical, as device architecture directly influences power consumption and switching speed at the circuit level. In particular, the balance between parasitic capacitance and resistance, key factors determining resistive‐capacitive (RC) delay, has not been thoroughly addressed. In this study, top‐gate CNT metal–oxide–semiconductor field‐effect transistors (MOSFETs) that employ a novel low‐κ extension doping technique are presented. This approach utilizes a stacked SiO_x_/AlF_x_ layer (κ = 3.9 and 2.5) to enhance device performance while minimizing parasitic capacitance between the top gate and contact metal. The proposed approach improves the driving current by 8.4 times and reduces the total resistance by 85% when compared with CNT MOSFETs with undoped extensions. The doping level is tunable from 0.5 nm^−1^ to 0.59 nm^−1^, with electrostatic doping driven by negative charges at the SiO_x_​/AlF_x_ interface. The benchmarking results demonstrate the record‐low κ‐value and physical thickness presented by the proposed approach, highlighting its potential for high‐performance, high‐speed applications.

## Graphical abstract

This study presents the first experimental demonstration of a low‐κ extension doping strategy for CNT MOSFETs using stacked SiO_x_/AlF_x_ layers. The proposed approach minimizes parasitic capacitance and reduces total resistance by 85%, significantly enhancing device performance. These findings establish a scalable, ultra‐thin doping method for high‐speed, energy‐efficient CNT‐based transistors.

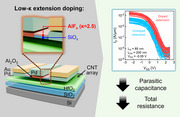

## 1 Introduction

With the advancement of semiconductor technology toward sub‐1 nm nodes, silicon‐based transistors face various challenges, with the most critical being the intrinsic degradation of carrier mobility in ultrathin‐body field‐effect transistors (FETs), such as FinFETs and nanosheet FETs.^[^ ^1^ ^]^ The channel thickness must be minimized to maintain a sharp switching behavior and suppress short‐channel effects. However, the mobility degradation becomes particularly severe when scaled below 5 nm, rendering it considerably challenging to sustain high performance at advanced technology nodes. Alternative channel materials are being actively analyzed to address this issue. Carbon nanotubes (CNTs) have emerged as the leading candidates for next‐generation, high‐performance, and energy‐efficient field‐effect transistors owing to their exceptional electrical properties and atomic‐scale dimensions.

The development of high‐density, aligned semiconducting CNT arrays represents a pivotal breakthrough in CNT technology, which can effectively overcome the limitations of non‐competitive drain currents associated with low‐density CNT channels in network configurations.^[^ ^2^ ^]^ These arrays demonstrate excellent driving currents exceeding 1 mA µm^−1^,^[^ ^2^, ^3^, ^4^, ^5^, ^6^, ^7^, ^8^, ^9^ ^]^ highlighting the considerable potential of CNT‐based transistors compared to conventional silicon‐based devices in terms of performance. Extensive research has been conducted on scaling down the physical dimensions of CNT‐based transistors. The channel lengths were successfully reduced below 15 nm while preserving the favorable transfer characteristics.^[^ ^2^, ^3^, ^4^ ^]^ Furthermore, the contact length was minimized to 10 nm, achieving a record‐low contact resistance of 6.5 kΩ per CNT.^[^ ^5^ ^]^ These breakthroughs highlight the considerable potential of carbon nanotube field‐effect transistors (CNFETs) as promising candidates for next‐generation nanoscale electronic devices. Additionally, the low fabrication temperature (<400 °C) renders CNT‐based transistors highly suitable for integration into the back‐end‐of‐line (BEOL) in monolithic three‐dimensional (M3D) technology platforms.

Despite the significant progress made in developing platforms for fabricating high‐performance CNT transistors, research on their device architecture remains limited. Although individual devices have achieved record‐breaking direct‐current (DC) performances, these achievements do not guarantee their practical relevance for industrial foundries or large‐scale production. Most state‐of‐the‐art CNFETs that exhibit excellent DC performance primarily depend on global gate configurations wherein the gate electrodes exhibit significant overlap with the source and drain electrodes.^[^ ^6^, ^7^, ^8^ ^]^ This architecture introduces considerable parasitic capacitance between the gate and source/drain electrodes, thereby severely limiting the device's competitiveness in terms of the operation frequency (speed) and power efficiency (energy).^[^ ^9^ ^]^ Furthermore, global‐gate designs exhibit degraded above‐threshold transitions, rendering them less competitive in advanced applications.^[^ ^10^ ^]^ This also applies to other emerging devices with advanced channel materials, such as transition metal dichalcogenides (TMDs).

Recent studies on reducing parasitic gate capacitance have focused on developing overlap‐free structures with air spacers between the top gate electrode and the contact metal, which effectively mitigate parasitic capacitance.^[^ ^11^ ^]^ However, the inclusion of undoped air spacers presents an additional extension resistance (R_EXT_) in series with the contact resistance (R_C_). This added resistance limits the overall device performance, particularly as the dimensions decrease. Consequently, achieving effective extension doping while maintaining a low parasitic capacitance is crucial for optimizing the power–speed of circuits comprising CNFETs.

Various approaches have been explored for p‐type extension doping by employing materials such as palladium (Pd) nanoparticles,^[^ ^12^ ^]^ tungsten oxides (WO_x_),^[^ ^13^ ^]^ and a stack of aluminum oxide (AlO_x_) beneath silicon nitride (SiN_x_).^[^ ^14^ ^]^ However, these methods face significant challenges, as they are either incompatible with very large‐scale integration (VLSI) processes or depend on high‐κ dielectrics with thicknesses exceeding 10 nm, thereby presenting increased parasitic capacitance and limited scalability. Recent simulation studies on doping strategies for carbon nanotube metal–oxide–semiconductor field‐effect transistors (MOSFETs) indicate that low‐κ extension doping (κ = 4) improves the energy‐delay product (EDP) by 1.8 times compared to high‐κ extension doping (κ = 18).^[^ ^15^ ^]^ However, the experimental validation of low‐κ doping in CNT‐based devices has not yet been performed.

In this study, we present a novel doping approach using stacked layers of silicon oxide (SiO_x_) and aluminum fluoride (AlF_x_) for extension doping, where SiO_x_ presents a dielectric constant of 3.9 and AlF_x_ serves as a low‐κ dielectric with a constant of 2.5. The proposed doping scheme utilizes ultra‐thin layers of SiO_x_ and AlF_x_, each with a thickness of 1 nm, demonstrating considerable potential for ultra‐scaled transistor applications. The doping strength can be tuned by adjusting the number of stacked layers, enabling doping levels from 0.5 to 0.59 nm^−1^.

The major contributions of extension doping for dense‐array CNT MOSFETs include (i) an 8.4‐fold increase in the driving current; (ii) reduced contact resistance, demonstrated by the output characteristics (I_D_‐V_DS_) transitioning from Schottky to ohmic behavior; and (iii) an 85% reduction in the total resistance, along with decreased variability. The benchmarking results demonstrate that this is the first experimental demonstration of a low‐κ doping strategy with the lowest physical thickness reported thus far. Additionally, the dielectric constant of AlF_x_ satisfies the 2031 International Roadmap for Devices and Systems (IRDS) requirements for device spacers, demonstrating its potential for future high‐performance and high‐speed electronic devices.

## 2 Results and Discussion

### 2.1 Low‐κ doping for CNT Electronics

**Figure 1:**
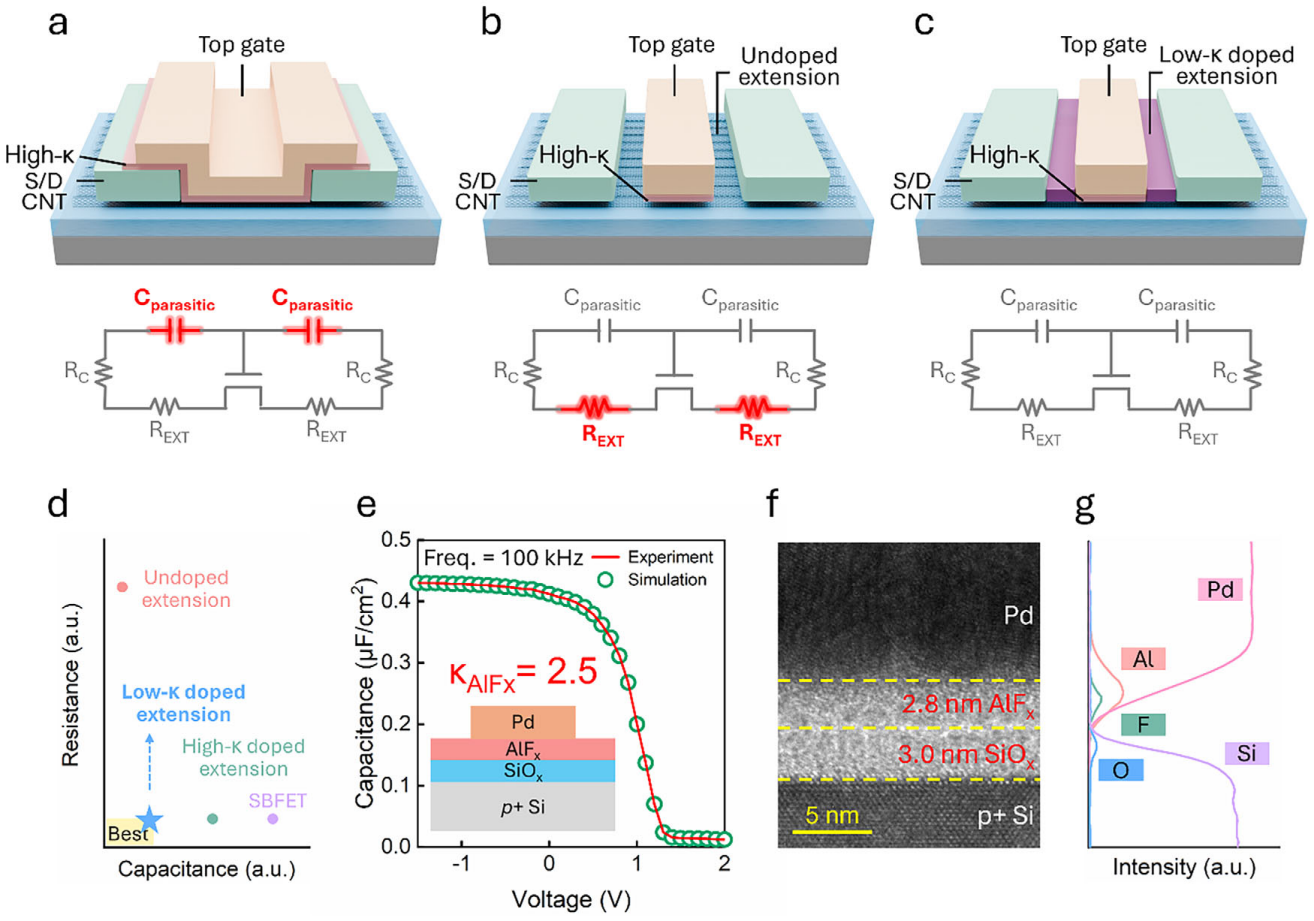
Schematics of CNT transistors with conventional device structures: a) Overlapped global gate architecture, also referred to as a Schottky barrier FET (SBFET); b) MOSFET with undoped extensions; and c) MOSFET with low‐κ doped extensions. The corresponding equivalent circuit models are depicted for each structure. d) Schematic of RC delay for various device architectures. e) Capacitance–voltage (C–V) characteristics of a silicon MOSCAP containing 3 nm of SiO_x_ and 2.8 nm of AlF_x_, where the red line represents experimental data and the green hollow dots represent the fitting results. (f) TEM image and (g) EDX line scan of the silicon MOSCAP.

**Figure** **1**a–c present a comparison of the commonly used CNT transistor architectures that have been reported recently, including the global gate Schottky barrier FETs (SBFETs) and MOSFETs with undoped and doped extensions. Undesired parasitic capacitance is observed in configurations where the top gate overlaps with the source and drain electrodes (Figure 1a), thereby increasing the RC delay (τ = CV/I). Conversely, while MOSFETs with undoped extensions (air spacers) mitigate the parasitic capacitance, extension resistance (R_EXT_) becomes crucial as the ungated extensions lack sufficient carrier density to ensure efficient charge transport. MOSFETs with low‐κ (κ < 3.9) doped extensions effectively address the challenges involved in achieving sufficient carrier density in the extensions while minimizing parasitic capacitance. Figure 1d depicts the relationship between the RC delay and device architecture.

In this study, we present a p‐type doping scheme using stacked layers of SiO_x_ and AlF_x_. Both materials are deposited within a high‐vacuum (<1×10^−7^ torr) e‐beam evaporator chamber at a low deposition rate (0.1 Å s^−1^) to accurately control the film thickness. The detailed deposition process is described in Section S1 (Supporting Information). Figure 1e depicts the capacitance–voltage (C–V) characteristics of a silicon MOS capacitor (MOSCAP) incorporating 3 nm SiO_x_ and 2.8 nm AlF_x_. Based on the best‐fit results from simulations, the dielectric constants of SiO_x_ and AlF_x_ were determined as 3.9 and 2.5, respectively. Figure 1f,g depicts the corresponding TEM image and EDX mapping of the MOSCAP. It is known that the dielectric constant of a material may vary depending on the substrate. The values for AlF_x_ and SiO_x_ extracted using CNT‐based capacitors are provided in Section S2 (Supporting Information).

**Figure 2:**
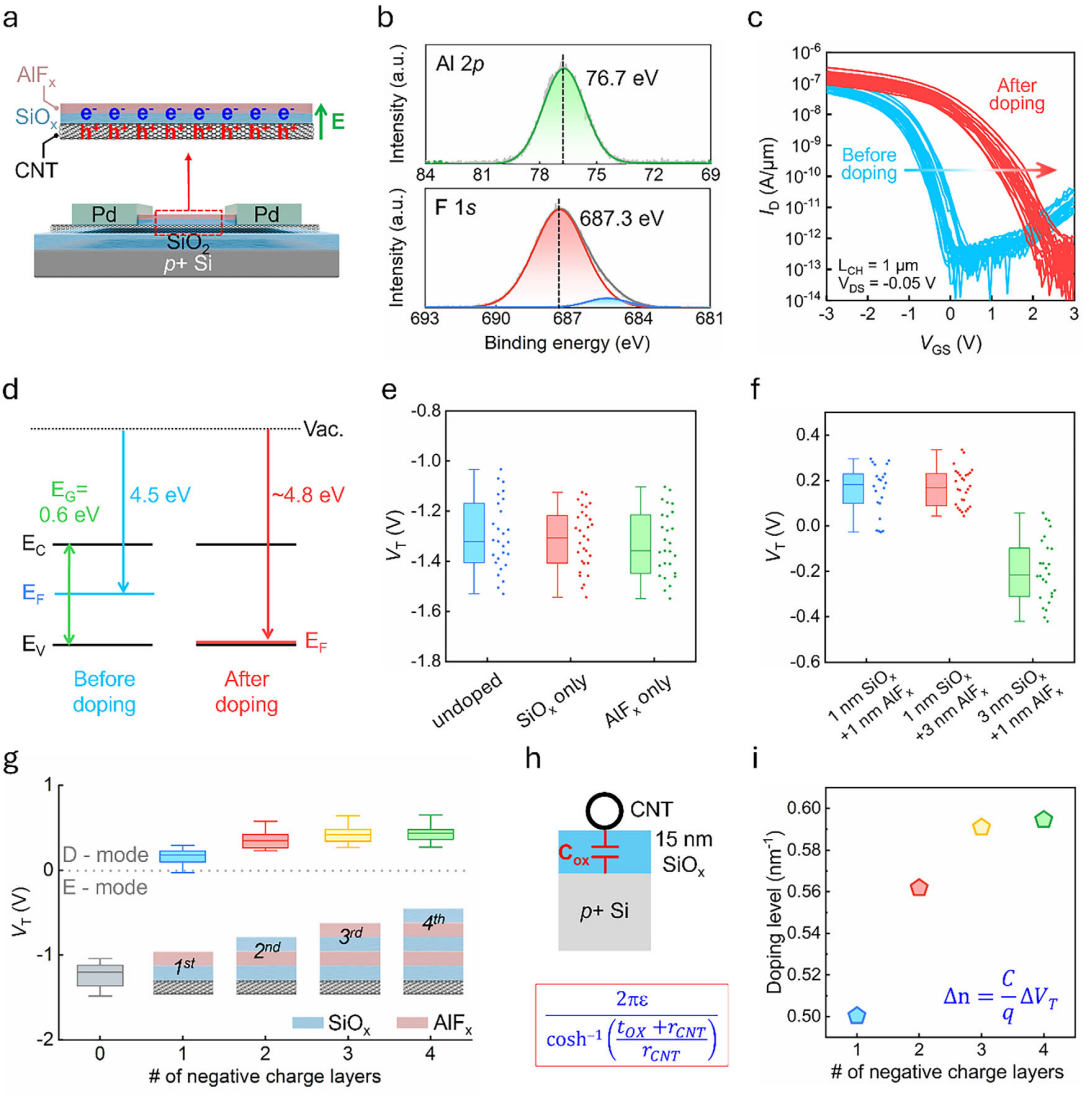
a) Schematic of a back‐gate CNFET with the entire channel covered by a 1 nm layer of SiO_x_ and a 1 nm layer of AlF_x_. Additional hole carriers in the CNT are induced by negative charges located at the SiO_x_/AlF_x_ interface. b) XPS spectra of Al 2p and F 1s for the AlF_x_ used in this study, with an Al‐to‐F ratio calculated as 1:2.8. c) Transfer curves of back‐gate CNFETs before and after coverage with a bilayer of SiO_x_ (1 nm) and AlF_x_ (1 nm). d) Band structure of the CNT before and after doping. e) Comparison of V_T_ for undoped CNFETs, CNFETs with the channel covered by 1 nm of SiO_x_ only, and those covered by 1 nm of AlF_x_ only. f) V_T_ comparison of CNFETs with channels covered by varying thicknesses of SiO_x_ and AlF_x_. g) V_T_ as a function of the number of negative charge layers. h) Schematic of the oxide capacitance of a CNT placed on a 15 nm SiO_x_ dielectric layer over a p^+^ silicon substrate. The equivalent capacitance per unit length (C′_ox_) is defined by the given equation, where *ε* represents the dielectric permittivity, *t_ox_* is the oxide thickness, and *r_CNT_* is the radius of the CNT. i) Doping level as a function of the number of negative charge layers.

P‐type doping was demonstrated by depositing a bilayer of SiO_x_ and AlF_x_ (1 nm each) on the channel of the back‐gate CNFETs (**Figure** **2**a). The channel CNTs are synthesized using the arc‐discharge method, with an energy bandgap of ≈0.6 eV. Material characterization details are provided in Section S3 (Supporting Information). The effectiveness of the doping can be efficiently evaluated through the threshold voltage shift (ΔV_T_) by covering the entire channel with the doping material,^[^ ^14^, ^16^ ^]^ which also enables the quantification of the doping level. Previous studies have reported a high concentration of negative charges (≈4×10^12^ cm^−2^) at the SiO_x_/AlF_x_ interface,^[^ ^17^ ^]^ attributed to fluoride (F) vacancies. In our case, these negative charges caused electrostatic doping, which induced excess hole carriers in the CNTs (Figure 2a).

Figure 2b depicts the XPS spectrum of AlF_x_. The F 1s core‐level spectrum exhibits a primary peak at 687.3 eV and a secondary peak at 685.1 eV. The secondary peak at a lower binding energy corresponds to the presence of minor species,^[^ ^18^ ^]^ such as the dangling bonds of F ions. The Al 2p spectrum peaks at 76.7 eV, which corresponds to Al–F bonding. The atomic ratio of Al to F was determined to be 1:2.8 by integrating the areas under the respective peaks.

After doping, the threshold voltage (extracted by maximum transconductance extrapolation method) shifted from −1.3 to 0.2 V. This shift is attributed to the generation of excess hole carriers within the channel, which reduces the barrier width at the contacts and requires a higher gate bias to impede the hole carrier transport from the contact to the channel. Although a “degenerate‐like” I_D_–V_GS_ curve, where the channel loses gate control ^[^ ^19^, ^20^ ^]^ as previously reported for MoO_x_‐based p‐type doping in CNTs, was not observed after doping, the proposed approach achieves sufficient doping strength. This is demonstrated by the transition of the device from the enhancement mode (E‐mode) to the depletion mode (D‐mode), indicating an adequate hole carrier density within the channel to enable conduction. Figure 2d depicts the corresponding band diagrams, where the Fermi level moved toward the valence band after doping. Notably, the overdrive current did not increase significantly after doping (Figure 2c), which can be attributed to the substantial resistance presented by the CNT–CNT junctions within the channel region,^[^ ^21^ ^]^ imposing a performance ceiling on the overall device. This limitation can be overcome by employing a dense array of CNT channels, as discussed in the following section.

We conducted a series of experiments using different doping schemes to verify that the doping effect originates from the interface, rather than from the bulk properties of the materials. First, we deposited single layers of SiO_x_ and AlF_x_ separately on the channel. The threshold voltage remained unchanged when compared with that of the undoped devices (Figure 2e). Subsequently, we independently increased the SiO_x_ and AlF_x_ thicknesses based on the initial 1 nm + 1 nm doping scheme. Increasing the thickness of AlF_x_ did not present a further shift in V_T_, whereas a thicker SiO_x_ layer led to a reduced V_T_ shift (Figure 2f). This reduction can be attributed to the increased distance between the negative charges and CNTs, which weakens the electrostatic doping effect. These results indicate that the doping effect arises from the interface between SiO_x_ and AlF_x_ rather than from the bulk charges of individual SiO_x_ or AlF_x_.

We demonstrated the tunability of the doping strength by increasing the number of stacked layers on the CNT, such as SiO_x_ ​/AlF_x_ ​/SiO_x_ ​, to introduce additional layers of negative charges (Figure 2g). Controlling the doping strength for CNT MOSFETs is vital as off‐state band‐to‐band tunneling (BTBT) leakage currents strongly correlate with the doping strength at extension.^[^ ^1^, ^10^, ^22^ ^]^ The threshold voltage shifted progressively toward positive values as the number of negative charge layers increased to four (SiO_x_ ​/AlF_x_ ​/SiO_x_ /AlF_x_ ​/SiO_x_, 1 nm each). This is attributed to the increasing total amount of negative charges, which induces a greater accumulation of hole carriers within the channel. The most significant V_T_ shift was observed when the first negatively charged layer was introduced, after which the shift became saturated with the addition of further layers. This saturation effect is attributed to the distance between the negative charges and the channel increasing with the number of subsequent layers, thereby reducing the incremental contribution of each additional layer to the accumulation of hole carriers. The doping level (Δn) can be estimated using the formula Δn = C/q*ΔV_T,_^[^ ^16^ ^]^ where C denotes the gate capacitance and q denotes the elementary charge. Contrary to 2D materials such as MoS_2_, for which the gate capacitance can be approximated using a parallel‐plate model, CNTs are 1D systems and must be considered as thin wires with a radius (r_CNT_) at a height (t_ox_) above the ground plane^[^ ^23^, ^24^, ^25^, ^26^ ^]^ (Figure 2h). Assuming that r_CNT_ = 0.75 nm, the doping level was quantified as 0.5 nm^−1^ for one negative charge layer, gradually increasing to 0.59 nm^−1^ for four negative charge layers (Figure 2i). The calculated doping level may have been slightly underestimated owing to the use of doped silicon as the gate electrode, which presents a lower conductivity than metals. The doping effect for larger bandgap HiPco CNT (bandgap = 0.85 eV) has also been studied in Sections S4 and S5 (Supporting Information).

### 2.2 Low‐κ Extension Doping for High‐Performance Dense Array CNT MOSFET

**Figure 3:**
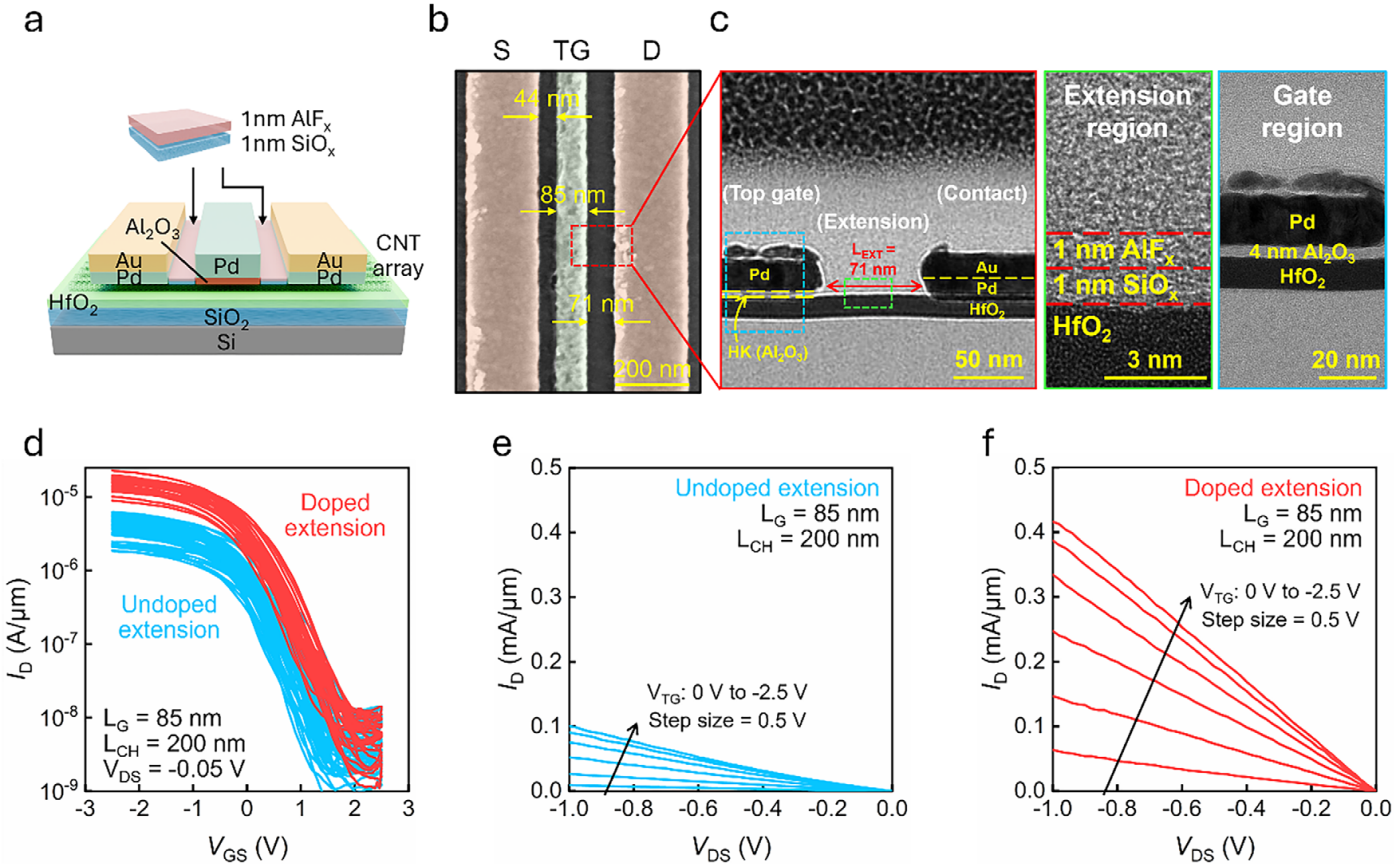
a) Schematic of CNT MOSFET with SiO_x_/AlF_x_ doped extensions. b) SEM image and c) TEM images of CNT MOSFET with SiO_x_/AlF_x_ doped extensions. d) Transfer curves of dense‐array CNT MOSFETs with and without extension doping. Output characteristics of dense‐array CNT MOSFETs with: e) undoped extension and f) doped extension.

To demonstrate the application of extension doping in high‐performance CNT MOSFETs, we use a dense‐array CNT channel with a density of ≈250 CNT µm^−1^, synthesized via the dimension‐limited self‐alignment (DLSA) method.^[^ ^27^ ^]^ The material characterization of the CNT array is presented in Section S6 (Supporting Information). The fabrication process involves defining the active area, followed by electron‐beam lithography (EBL) to pattern the contact regions. Palladium (Pd)/ gold (Au) was then deposited as the contact metals through e‐beam evaporation. The gate region was defined using EBL to form an air‐exposed spacer. Subsequently, a 4 nm layer of AlO_x_​ was deposited at 90 °C through atomic layer deposition (ALD) to serve as the gate dielectric, and Pd was deposited as the gate metal. A liftoff process was employed to complete the fabrication, presenting a CNT MOSFET featuring an air‐exposed spacer. Lastly, SiO_x_ ​and AlF_x_​ were deposited through e‐beam evaporation to implement extension doping; **Figure** **3**a depicts the device structure. SEM demonstrates that the gate length is 85 nm and the spacer regions are 44 and 71 nm (Figure 3b), with the corresponding TEM images shown in Figure 3c. The TEM image of the extension region without visual guidance is demonstrated in Figure S7 (Supporting Information). With localized extension doping, the maximum drain current (I_max_) is enhanced by a factor of three when compared with the CNT MOSFET with undoped extensions (Figure 3d). This improvement is primarily attributed to the reduction in both the extension resistance (R_EXT_) and contact resistance (R_C_), as doping near the contact region decreases the Schottky barrier width.^[^ ^28^ ^]^ The subthreshold swing (SS) remained unchanged after extension doping (Figure S8, Supporting Information). Figure 3e,f depicts the output characteristics of devices with and without extension doping, where the maximum output current at V_DS_ = −1 V increases from 0.1 mA µm^−1^ to over 0.4 mA µm^−1^. Furthermore, the Schottky‐like behavior observed at low V_DS_ in MOSFETs with undoped extensions was no longer present after doping, indicating that extension doping significantly reduced the contact resistance and enhanced the charge injection efficiency.

**Figure 4:**
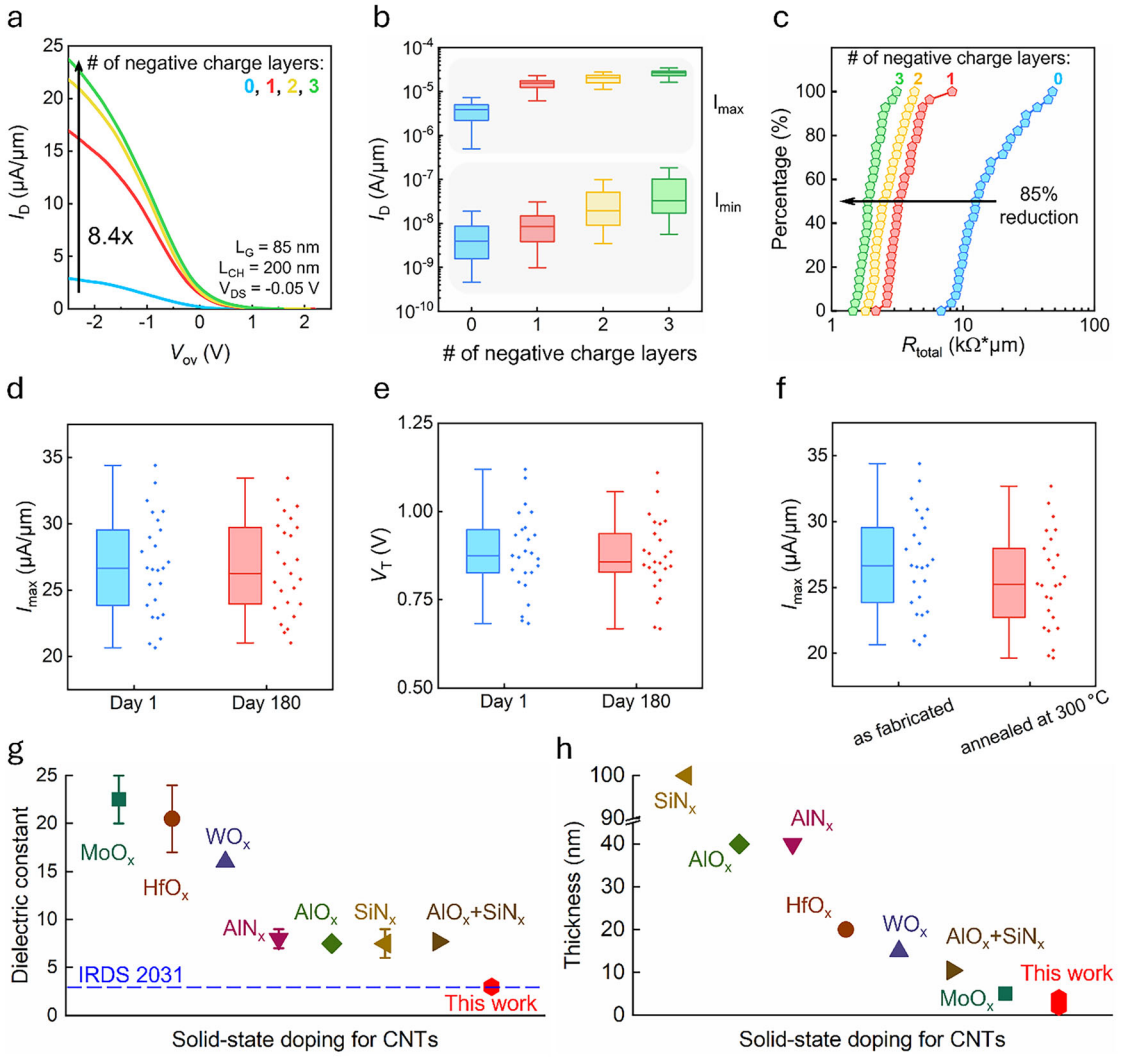
a) Overdrive current characteristics of dense‐array CNT MOSFETs with extensions doped using varying numbers of doping layers. b) Maximum current (I_max_) and minimum current (I_min_) as functions of the number of doping layers. c) CDF plot of total resistance for CNT MOSFETs with extensions doped using different numbers of doping layers. Stability tests were conducted 180 days post‐fabrication, showing d) I_max_ and e) threshold voltage. f) Comparison of I_max_ after annealing at 300 °C for 60 minutes. g) Benchmarking of reported solid‐state doping techniques for CNTs based on dielectric constant, with this work achieving the lowest κ‐value (2.5) and meeting the IRDS 2031 requirements for spacer materials. h) Benchmarking of reported solid‐state doping techniques for CNTs based on physical thickness, with this work demonstrating the thinnest doping layers (2–4 nm) reported to date.

Subsequently, we modulated the doping level by increasing the number of negative charge layers at the extensions (up to a maximum of three layers) to achieve stronger doping, as discussed in the previous section. Increasing the doping level further enhances the driving current (**Figure** **4**a). We selected a bias of V_DS_ = −0.05 V for this analysis to minimize the influence of additional band bending at the drain under high V_DS_. The performance improvement begins to saturate as the negative charges are positioned further from the CNT within the spacer region, which concurs with the observed trend of the threshold voltage shift in back‐gated CNFETs. The comparison of the driving current across various device structures, including MOSFETs with doped extensions and SBFETs, is provided in Section S9 (Supporting Information). Figure 4b depicts the relationship between the maximum drain current (I_max_) and minimum current (I_min_) across various doping levels. Both I_max_ and I_min_ increase with the increasing doping levels. The I_min_ value of CNT MOSFETs is primarily governed by BTBT, which is significant due to their relatively small bandgap (≈0.6 eV) and is strongly dependent on the doping level at extension.^[^ ^1^ ^]^ The BTBT leakage currents can be effectively suppressed by increasing the bandgap.^[^ ^10^, ^22^, ^29^ ^]^ Figure 4c depicts the cumulative distribution function (CDF) plot of the total device resistance, which reveals a reduction of 85% in the median resistance under the strongest doping conditions demonstrated in this study. Additionally, the distribution of the total resistance converges after extension doping, indicating that this doping method effectively improves the device performance across a range of initial resistances. This improvement is crucial for devices with a higher initial contact resistance, which can arise from bandgap variations among the individual CNTs or metal work‐function differences for different metal surfaces facing the CNTs. The parasitic resistance (R_P_ = 2R_C_ + 2R_EXT_) has been extracted and is presented in Section S10 (Supporting Information).

Lastly, we tested the samples stored in air for 180 days to evaluate the stability of this doping method. The on‐current and threshold voltage remained unchanged, indicating excellent air stability and resistance to rapid degradation (Figure 4d,e). The thermal stability of the annealed samples was also evaluated in a nitrogen‐filled glovebox at 300 °C for 60 minutes. The on‐current exhibited only a slight decrease of 5% after annealing, suggesting strong thermal resilience (Figure 4f). Further improvements are expected with appropriate device passivation.

We benchmarked our doping strategy against the recently reported solid‐state doping approaches for CNTs,^[^ ^13^, ^14^, ^19^, ^29^, ^30^, ^31^, ^32^ ^]^ focusing on the dielectric constant and thickness of the doping material (Figure 4g,h). In this study, we reported the only solid‐state low‐κ material with a dielectric constant of 2.5. Low‐κ materials are crucial not only for spacers in front‐end‐of‐line (FEOL) transistors but also for back‐end‐of‐line (BEOL) interconnect stacks, where they are essential for sustaining performance gains, such as faster switching speeds, reduced power consumption, and minimized crosstalk. Furthermore, the proposed doping method satisfied the IRDS 2031 target for the spacer dielectric constant. In addition to its dielectric properties, the proposed approach presents the most competitive doping thickness, addressing the stringent spatial constraints of advanced technologies as device dimensions continue to scale down laterally and vertically. Doping materials with thicknesses exceeding 10 nm are incompatible with such constraints, rendering the proposed method particularly suitable for next‐generation transistor architectures. An additional benchmark comparing this work with the reported CNT MOSFETs with doped extensions is provided in Section S11 (Supporting Information).

## 3 Conclusion

In this study, we present a novel low‐k extension doping approach for top‐gate CNT MOSFETs. The proposed doping method satisfies the IRDS 2031 requirement for spacer dielectric constants. The electrostatic doping mechanism is verified, facilitated by negative charges at the SiO_x_/AlF_x_ interface, with tunable doping strengths ranging from 0.5 to 0.59 nm^−1^. Implementing this doping scheme in the extension region of dense‐array CNT MOSFETs enhances the driving current by a factor of 8.4 and reduces the device‐to‐device variability. In future work, we aim to expand upon this study by analyzing n‐type doping strategies to develop a complementary approach for high‐performance, high‐speed CNT NMOS devices, facilitating their integration into complementary logic circuits.

## Conflict of Interest

The authors declare no conflict of interest.

## Supporting information



Supporting Information

## Data Availability

The data that support the findings of this study are available in the supplementary material of this article.
