# Supplementary material for: Low‐κ Extension Doping for High‐Performance Carbon Nanotube Transistors: Toward High‐Speed, Energy‐Efficient Electronics
Source: Adv Sci (Weinh). 2025 Jun 5;12(33):e05543. doi: 10.1002/advs.202505543 (PMC12412536; doi:10.1002/advs.202505543)
Supplement: Supplementary file 1 — Supporting Information [file ADVS-12-e05543-s001.docx]

Supporting Information

Title

**Low-κ Extension Doping for High-Performance Carbon Nanotube Transistors: Toward High-Speed, Energy-Efficient Electronics**

Hsin-Yuan Chiu^1^, Chen-Han Chou^1^, Guan-Zhen Wu^1^, Han-Yi Huang^1^, Bo-Heng Liu^2^, Chi-Chung Kei^2^ and Chao-Hsin Chien^1*^

*^1^ Institute of Electronics, National Yang Ming Chiao Tung University, Hsinchu 30010, Taiwan*

*^2^ Taiwan Instrument Research Institute, National Applied Research Laboratories, Hsinchu 30076, Taiwan*

E-mail: [chchien@nycu.edu.tw](mailto:chchien@nycu.edu.tw)

1. **The deposition of SiO_x_ and AlF_x_ layers.**

Both the SiO_x_ and AlF_x_ layers were deposited using e-beam evaporation. A transparent SiO_x_ target (1–3 mm in size) and a white pellet AlF_x_ target (1–3 mm in size) were used for the respective depositions. Due to the low melting point of AlF_x_, the e-beam power was carefully regulated—maintaining a voltage of 8 kV and a current below 10 mA—to ensure a stable deposition rate of 0.1 Å/s. All depositions were performed under high-vacuum conditions, with the chamber pressure maintained below 1 × 10⁻^7^ torr, ensuring high film purity and stoichiometric control.

1. **Extraction of dielectric constants of AlF_x_ and SiO_x_ using CNT-based capacitors.**


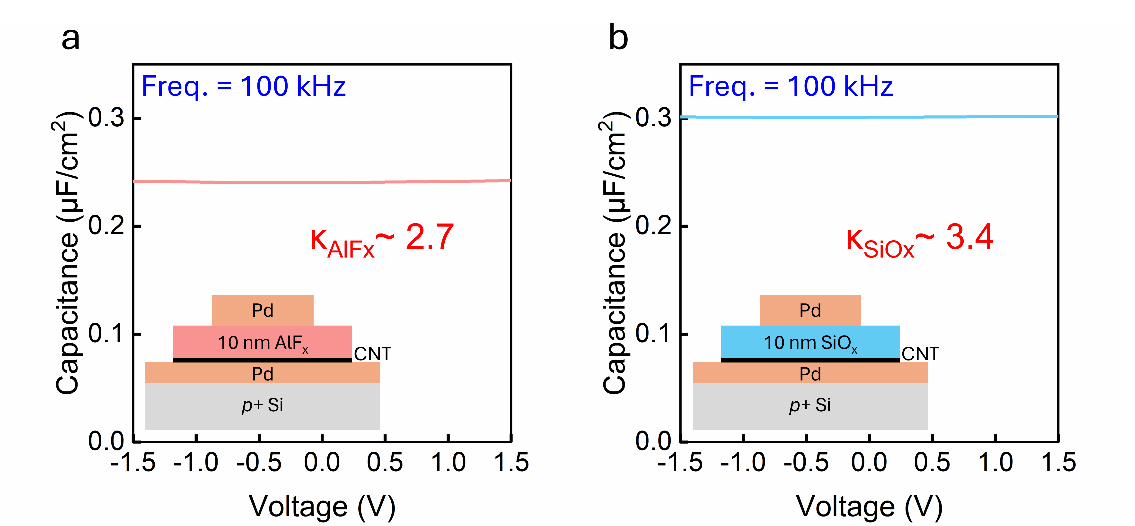
We fabricated metal–insulator–semiconductor–metal (MISM) structures with the configuration Pd/AlFₓ (or SiOₓ)/CNT/Pd to enable direct extraction of the dielectric constants using CNT-based capacitors. A schematic illustration of the MISM structure is provided in **Figure S2**. Based on the measured capacitance, the dielectric constants of AlFₓ and SiOₓ were determined to be 2.7 and 3.4, respectively.

**Figure S2.** Schematic illustration of the CNT-based capacitor structures and dielectric constant extraction for (a) AlFₓ and (b) SiOₓ.

1. **Material characterization of low-density arc-discharge CNT network.**

The Atomic Force Microscope (AFM) image of the arc-discharge CNT network is shown in **Figure S3a**, with a density of approximately 25 CNTs/μm. The absorption spectrum of arc-discharge CNTs is presented in **Figure S3b**, showing no detectable metallic M_11_ peak. The corresponding Raman spectrum is shown in **Figure S3c**, with a D/G ratio of 1:16.

**
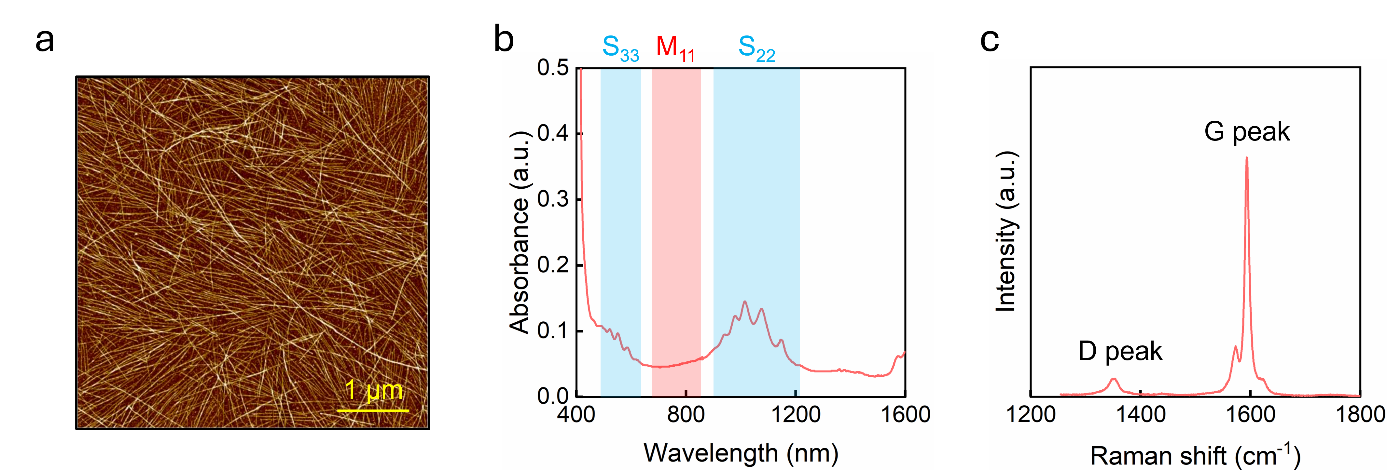
**

**Figure S3.** (a) AFM image, (b) absorption spectrum, and (c) Raman spectrum of the arc-discharge CNTs used in this work.

1. **Low-κ doping for large bandgap HiPco CNFETs.**

Beyond demonstrating low-κ doping for small-bandgap arc-discharge CNTs (E_G_ ~ 0.6 eV), our SiO_x_/AlF_x_ doping strategy is also applicable to large-bandgap HiPco CNTs (E_G_ ~ 0.85 eV). After doping, the threshold voltage shifts from −1.7 V to −1.3 V (**Figure S4a**). The ΔV_T_ is smaller than that observed in arc-discharge CNTs due to the larger bandgap, which results in a higher Schottky barrier at the contact/CNT interface. As the number of negative charge layers increases, the threshold voltage shifts further toward positive values before saturating, as shown in **Figure S4b**, consistent with the trend observed in arc-discharge CNTs (**Figure 2g**). The material characterization of the HiPco CNTs used in this work is presented in **Supplementary Section 5**.


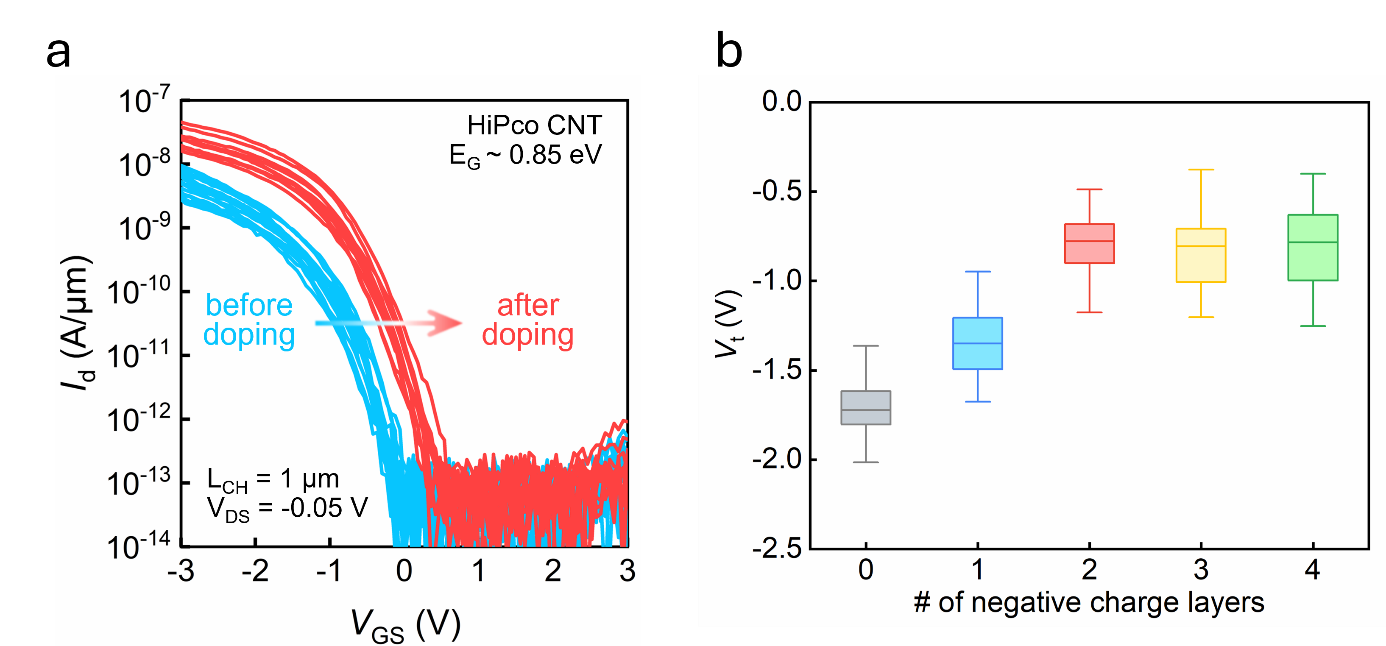


**Figure S4.** (a) Transfer curves of back-gate HiPco CNFETs before and after coverage with a bilayer of SiO_x_ (1 nm) and AlF_x_ (1 nm). (b) Threshold voltage as a function of the number of negative charge layers.

1. **Material characterization of low-density HiPco CNT network.**

The AFM image of the HiPco CNT network is shown in **Figure S5a**, with a density of approximately 12 CNTs/μm. The absorption spectrum of HiPco CNTs is presented in **Figure S5b**. The corresponding Raman spectrum is shown in **Figure S5c**, with a D/G ratio of 1:7. CNTs synthesized using the HiPco (High-Pressure Carbon Monoxide) method typically exhibit higher defectivity compared to those produced by the arc-discharge method.


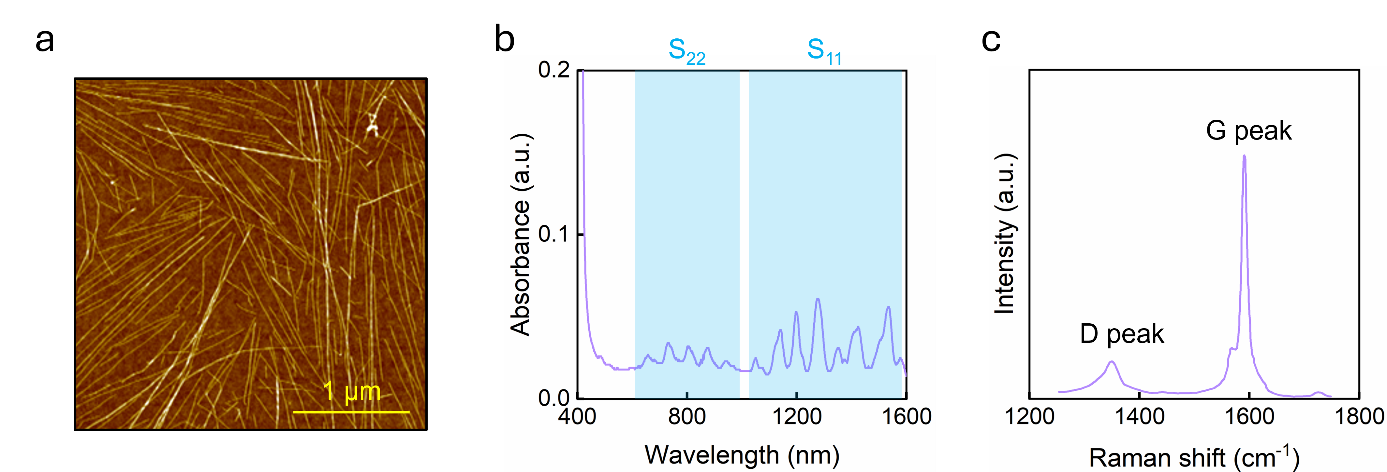


**Figure S5.** (a) AFM image, (b) absorption spectrum, and (c) Raman spectrum of the HiPco CNTs used in this work.

1. **Material characterization of the high-density CNT array.**

The high-density CNT array is prepared using the dimension-limited self-alignment (DLSA) method. This technique enables a significantly higher driving current compared to single-CNT FETs or CNT network FETs, owing to its high CNT density of approximately 250 CNTs/µm. The AFM image in **Figure S6a** illustrates the highly aligned CNT array, while the polarized Raman spectrum in **Figure S6b** confirms the strong alignment of the CNTs.


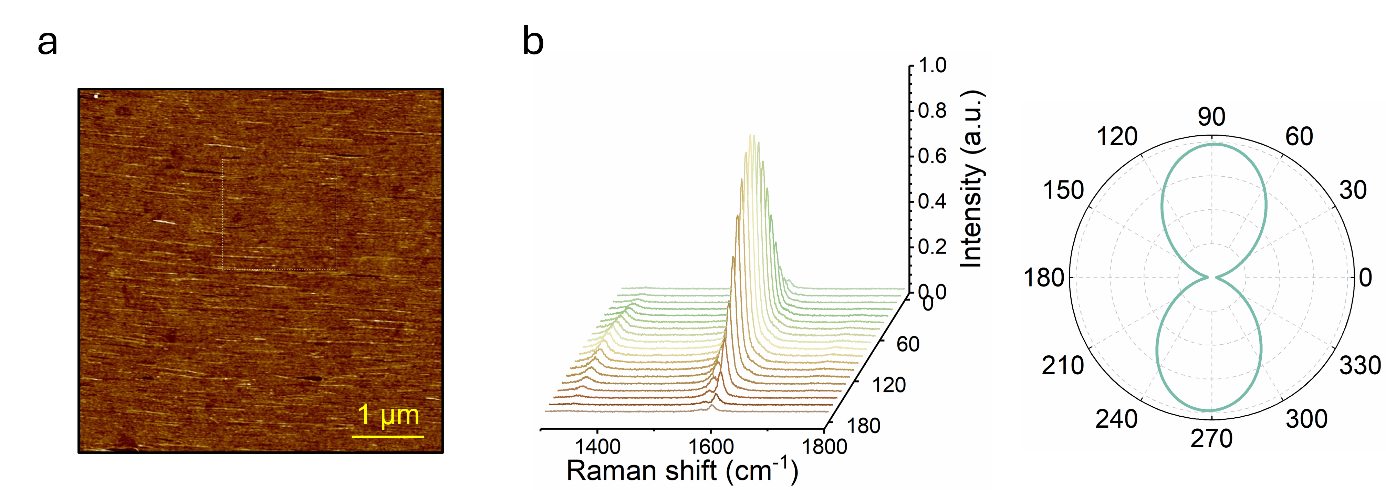


**Figure S6.** (a) AFM image, and (b) polerized Raman spectrum of the high-density CNT array used in this work.

1. **TEM image of the extension region doped by SiO_x_ and AlF_x_.**


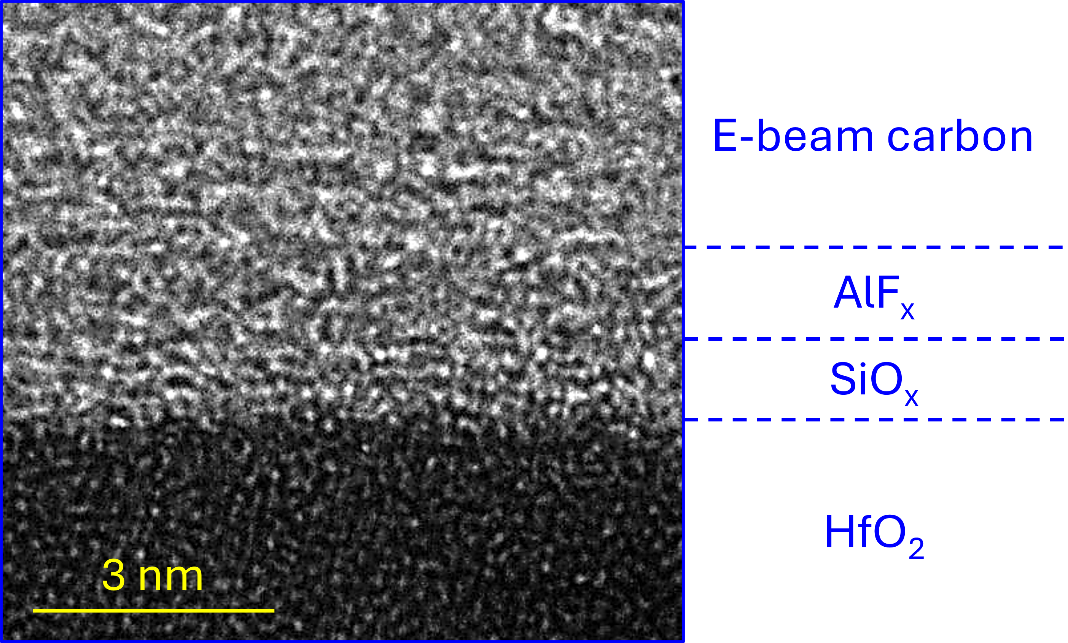


**Figure S7.** TEM image of the extension region doped by SiO_x_ and AlF_x_.

1.
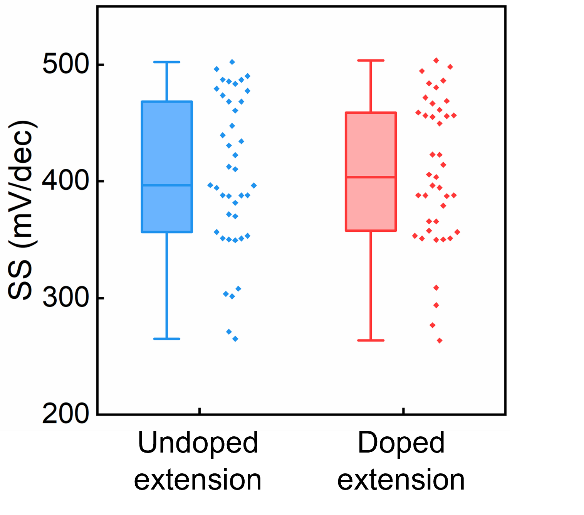
**Subthreshold swing comparison between CNT MOSFETs with undoped and doped extensions.**

**Figure S8.** Box plot comparison of the subthreshold swing for CNT MOSFETs with undoped (blue) and doped (red) extensions. The results indicate that extension doping does not degrade SS, maintaining similar switching characteristics.

1. **Driving current comparion among different structures.**

We fabricated self-aligned top-gate FETs (referred to as SBFETs) with a channel length of 200 nm, consistent with the CNT MOSFETs presented in this work to enable a fair performance comparison. A schematic illustration of the SBFET structure and the corresponding I_D_–V_GS_ transfer characteristics are provided below.


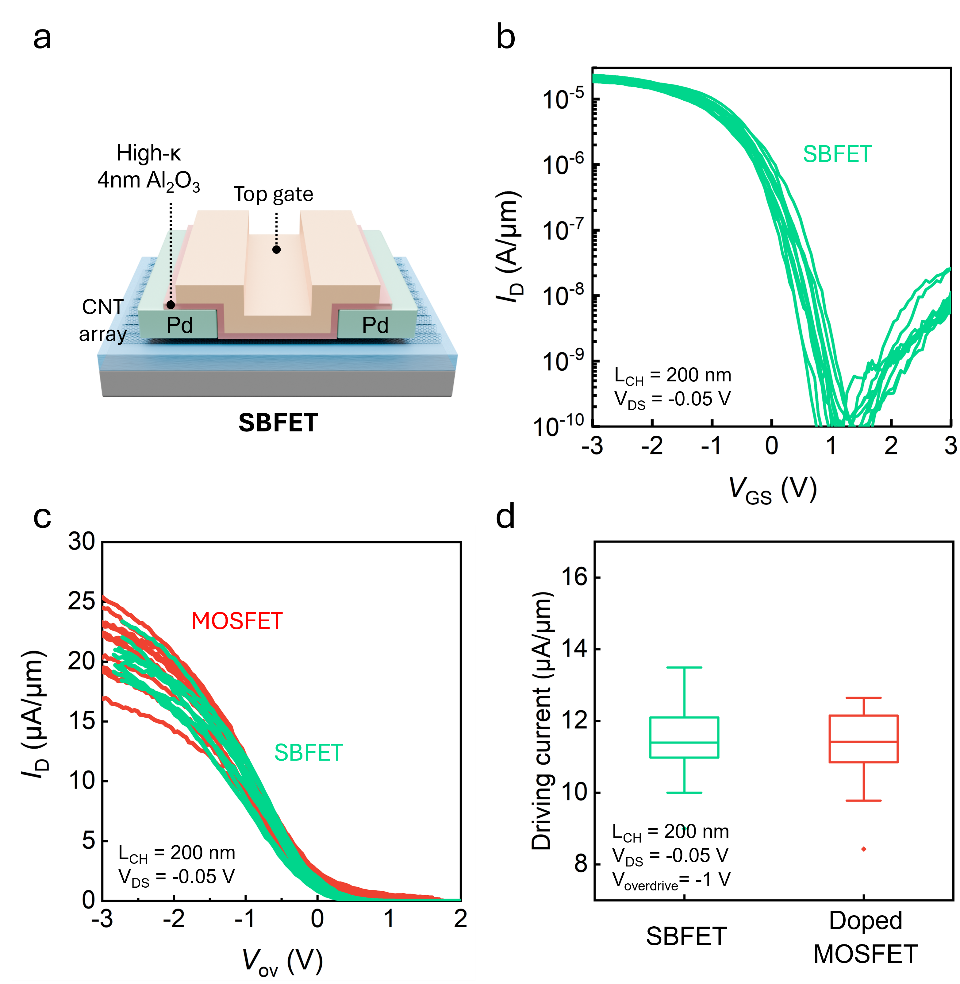
The results show that CNT MOSFETs with doped spacers exhibit a comparable median driving current to the self-aligned devices, indicating that the MOSFET structure—when combined with proper extension doping—maintains high performance. This confirms the effectiveness of the proposed low-κ doping scheme in minimizing extension resistance (R_EXT_) while preserving low parasitic capacitance.

**Figure S9.** (a) Schematic illustration of a CNT SBFET with a channel length (L_CH_) of 200 nm. (b) Transfer characteristics (I_D_–V_GS_) of CNT SBFETs. (c) Comparison of overdrive current between MOSFETs with doped extensions and SBFETs. (d) Statistical distribution of the driving current for both structures.

1. **Parasitic resistance (R_P_) extraction.**


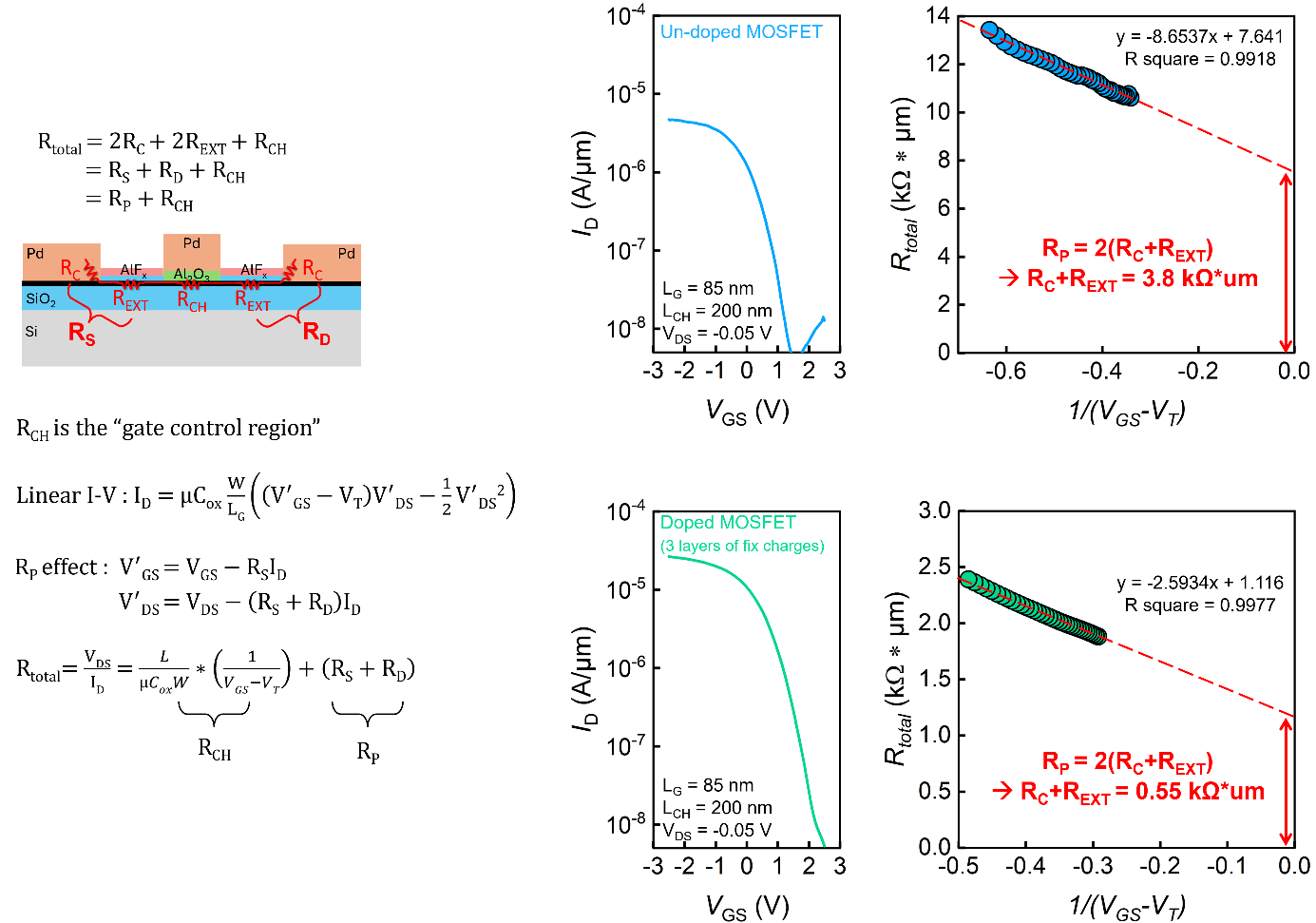


**Figure S10.** Extraction method and results for parasitic resistance. After applying extension doping with three layers of negative fixed charges, R_P_ is reduced from 7.6 kΩ·μm to 1.16 kΩ·μm, corresponding to an ~85% reduction.

1.
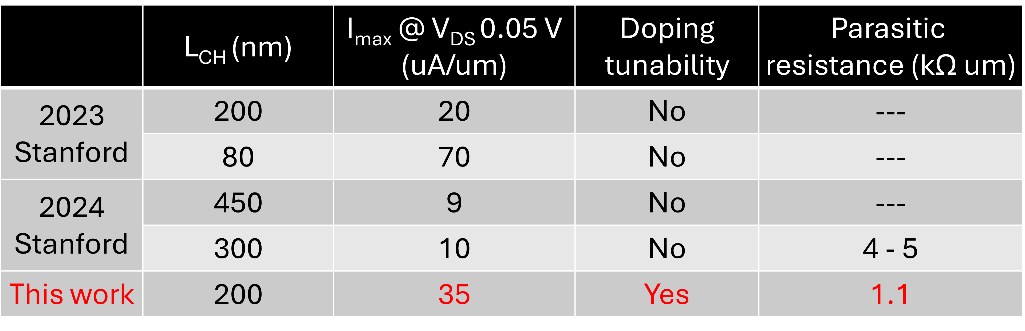
**Benchmark comparing this work with the reported CNT MOSFETs with doped extensions^1,2^.**

(1) Li, S.; Chao, T. A.; Gilardi, C.; Safron, N.; Su, S. K.; Zeevi, G.; Bechdolt, A. D.; Passlack, M.; Oberoi, A.; Lin, Q.; et al. High-performance and low parasitic capacitance CNT MOSFET: 1.2 mA/μm at VDS of 0.75 V by self-aligned doping in sub-20 nm spacer. In 2023 International Electron Devices Meeting (IEDM), 9-13 Dec. 2023, 2023; pp 1-4. DOI: 10.1109/IEDM45741.2023.10413827.

(2) Li, S.; Zhong, D.; Gilardi, C.; Safron, N.; Chao, T. A.; Zeevi, G.; Rijs, S. V.; Bechdolt, A. D.; Passlack, M.; Pitner, G.; et al. ISO-Performance N-Type and P-Type MOSFETs on Densely Aligned CNT Array Enabled by Self-Aligned Extension Doping with Barrier Booster. In 2024 IEEE International Electron Devices Meeting (IEDM), 7-11 Dec. 2024, 2024; pp 1-4. DOI: 10.1109/IEDM50854.2024.10873418.
